# Tetraniliprole Triggers Transgenerational Hormesis in an Invasive Insect Herbivore: Molecular and Biological Insights

**DOI:** 10.3390/insects16101073

**Published:** 2025-10-21

**Authors:** Farman Ullah, Zeeshan Ullah, Ali Güncan, Guru-Pirasanna-Pandi Govindharaj, Hina Gul, Prabhu Prasanna Pradhan, Ghulam Murtaza, Xiaowei Li, Nicolas Desneux, Yaobin Lu

**Affiliations:** 1 Institute of Bio-Interaction, Xianghu Laboratory, Hangzhou 311258, China; farmanullah787@gmail.com; 2 State Key Laboratory for Quality and Safety of Agro-Products, Key Laboratory of Biotechnology in Plant Protection of MOA of China and Zhejiang Province, Institute of Plant Protection and Microbiology, Zhejiang Academy of Agricultural Sciences, Hangzhou 310021, China; gulhina680@gmail.com (H.G.); lixiaowei1005@163.com (X.L.); 3 Department of Entomology, Abdul Wali Khan University Mardan, Mardan 23200, Pakistan; zeeshanullah212@gmail.com; 4 Department of Plant Protection, Faculty of Agriculture, Ordu University, 52200 Ordu, Turkey; guncan.ali@gmail.com; 5 Plant Quarantine Division, ICAR-National Bureau of Plant Genetic Resources, Regional Station, Hyderabad 500030, India; guruagri@gmail.com; 6 ICAR-Central Rice Research Institute, Cuttack 753006, India; prabhuprasannapradhan@gmail.com; 7 State Key Laboratory of Plant Diversity and Specialty Crops, South China Botanical Garden, Chinese Academy of Sciences, Guangzhou 510650, China; murtazabwn54@gmail.com; 8 Université Côte d’Azur, INRAE, CNRS, UMR ISA, 06000 Nice, France

**Keywords:** diamide, *Tuta absoluta*, hormesis, sublethal effects, life table, insecticide resistance

## Abstract

The South American tomato pinworm, *Tuta absoluta* (Meyrick), is among the most destructive invasive pests of tomato globally. The diamide insecticide tetraniliprole is increasingly used for its management. This study examines the sublethal effects of tetraniliprole on *T. absoluta* larvae, with a focus on its transgenerational impacts. Bioassays demonstrated that tetraniliprole was highly toxic to third-instar *T. absoluta* larvae, with an LC_50_ of 0.029 mg/L. Sublethal (LC_10_) and low lethal concentrations (LC_30_) were used to investigate their impact on developmental, reproductive, and population parameters across two subsequent generations (F_1_ and F_2_). In the parental (F_0_) generation, exposure to tetraniliprole at both concentrations significantly prolonged larval and pupal durations and reduced adult longevity and fecundity. In both F_1_ and F_2_ generations, concentration-dependent effects were observed—LC_10_ accelerated development and enhanced fecundity and population growth, indicative of a hormetic response, whereas LC_30_ delayed development and suppressed reproduction and survival. Life table analyses revealed significant changes in the *r*, *λ*, and *T*, particularly under LC_30_. Additionally, the RT-qPCR analysis revealed the downregulation of development and reproduction-related genes (*Vg*, *VgR*, and *JHBP*) in the F_0_ generation following exposure to tetraniliprole (LC_10_ and LC_30_). In contrast, these genes were upregulated in the progeny generations (F_1_ and F_2_) at LC_10_. Furthermore, the overexpression of key detoxification genes, particularly *CYP4M116* and *CYP6AW1*, persisted across all three generations. Taken together, these findings reveal a substantial risk of unintended population resurgence (hormesis effects) at sublethal concentrations, underscoring the importance of integrating transgenerational consequences into insecticide resistance management programs for sustainable control of this key insect pest.

## Simple Summary

This study investigated the sublethal effects of tetraniliprole on the invasive tomato pest, *Tuta absoluta*. Although tetraniliprole caused high toxicity in third-instar larvae, sublethal (LC_1_0) and low lethal (LC_30_) concentrations had complex effects across generations. The directly exposed generation (F_0_) experienced decreased development and reproduction. However, its offspring (F_1_ and F_2_) showed a dual response: the LC_10_ promoted faster development and increased population growth (hormetic-like effects), while the LC_30_ continued to cause negative effects. These changes were linked to altered expression of key genes involved in reproduction, development, and detoxification. The results highlight a significant risk of unintended population resurgence at sublethal concentrations and emphasize the importance of considering these transgenerational effects in resistance management strategies.

## 1. Introduction

The South American tomato pinworm, *Tuta absoluta* (Meyrick) (Lepidoptera: Gelechiidae), is recognized as one of the most devastating insect pests of tomato crops [1,2]. Since its emergence in South America, *T. absoluta* has rapidly expanded its geographic range and now invades over 90 countries worldwide [2,3]. In addition to tomatoes, this invasive pest threatens other economically significant solanaceous crops such as potato, eggplant, pepper, and tobacco [4,5]. Its high reproductive capacity, cryptic larval behavior, and resistance to conventional insecticides contribute to its invasiveness and the extensive damage it causes to crops [6,7]. Infestations by *T. absoluta* result in substantial yield losses, reduced fruit quality, and increased pest management costs [2,8,9].

Although several eco-friendly approaches are available [10,11], chemical insecticides remain widely used against this pest due to their rapid results. Tetraniliprole, a third-generation anthranilic diamide insecticide, acts by targeting the ryanodine receptors in insects, disrupting calcium ion regulation and ultimately causing paralysis and death [12]. This mode of action is effective against a broad spectrum of insect pests, including Lepidoptera, Coleoptera, and Diptera, with the added advantage of minimal toxicity to non-target mammals [13,14,15]. However, insecticides are known to degrade over time following initial field application [16], potentially exposing arthropod populations to sublethal doses or concentrations [17]. Such exposures can induce a variety of physiological and behavioral changes, affecting insect survival, development, reproduction, and even resistance evolution [18,19,20,21]. Interestingly, sublethal doses or concentrations can sometimes paradoxically enhance certain biological traits, such as reproduction, a phenomenon known as hormesis [22,23].

Sublethal effects can also impact insect reproductive systems at the molecular level. For example, vitellogenin (*Vg*), the precursor of vitellin (*Vn*), and its receptor (*VgR*), which facilitates *Vg* transport into oocytes, play crucial roles in insect fecundity and are often disrupted under insecticidal stress [24,25]. These disruptions may affect both directly exposed individuals and their progeny, highlighting the need to assess both direct and transgenerational impacts of insecticides. Beyond reproductive genes, cytochrome P450 monooxygenases (CYPs) are central to insecticide detoxification and have long been implicated in resistance development. The upregulation of cytochrome P450 enzymes during resistance development in *T. absoluta* leads to decreased population growth rates since it creates substantial fitness costs, manifesting when insecticide pressure disappears. By investing metabolic resources in resistance mechanisms, insects suffer fitness costs that trigger reduced fecundity stress during development and growth, impairing total fitness [19,20,21].

Life table analyses offer a comprehensive framework to assess the cumulative effects of various stressors on insect populations, including survival, development, longevity, and reproductive potential [26,27]. Unlike traditional female-only life tables [28], the age-stage, two-sex life table approach incorporates both sexes and accounts for individual variability in developmental rates, thereby providing more accurate and holistic insights into population dynamics [27,29].

Given the widespread use of tetraniliprole and the growing concern about its sublethal impacts, it is essential to evaluate its effects not only on exposed individuals but also on their progeny. The current study investigates the sublethal and low-lethal effects of tetraniliprole on *T. absoluta* by assessing key biological traits across three generations (F_0_, F_1_, and F_2_) using an age-stage, two-sex life table approach. The findings from this study will enhance our understanding of tetraniliprole’s impact on population fitness and reproductive potential and offer valuable insights for the sustainable management of *T. absoluta*.

## 2. Materials and Methods

### 2.1. Insect

The initial colony of *T. absoluta* was established from larvae collected in tomato fields in Yuxi (24.3473° N, 102.5274° E), Yunnan Province, China, in June 2018. The population was subsequently maintained under laboratory conditions for several years without exposure to any insecticides to ensure a susceptible baseline strain. Rearing was conducted on pesticide-free tomato plants under controlled environmental conditions: 25 ± 1 °C, 60 ± 5% relative humidity, and a photoperiod of 16:8 h (light/dark).

### 2.2. Toxicity Bioassays

Adult *T. absoluta* were introduced onto fresh, pesticide-free tomato plants for a 12 h oviposition period. Following egg deposition, the infested plants were transferred to clean rearing cages to allow for hatching and larval development. This protocol ensured uniformity in larval age and developmental stage, with third-instar larvae subsequently selected for bioassay experiments. These larvae were designated as the parental (F_0_) generation. The toxicity of tetraniliprole against third-instar *T. absoluta* larvae was evaluated using the standardized leaf-dip bioassay method described [30], under the same controlled laboratory conditions. A technical grade (95% purity) formulation of tetraniliprole was used to prepare a stock solution in analytical-grade acetone, which was serially diluted in distilled water containing 0.05% Triton X-100 (Sangon Biotech Co., Ltd., Shanghai, China) to obtain seven concentrations (0.0078, 0.0156, 0.0312, 0.0625, 0.125, 0.25, and 0.5 mg/L). The control treatment consisted of distilled water containing 0.05% Triton X-100 alone. Fresh tomato leaves were individually immersed in each insecticide concentration for 15 s and then air-dried at room temperature for 1–2 h. To maintain leaf turgidity, the petioles were wrapped in moistened cotton wool. Upon air-drying, treated leaves were placed in Petri dishes (diameter 9 cm × height 1.5 cm) lined with filter paper. For each replication under both treatment and control, 20 third-instar larvae were carefully transferred onto the treated leaves. Each treatment was replicated three times. Larval mortality was recorded 48 h post-treatment. Larvae unresponsive to gentle probing with a fine brush were recorded as dead. The mortality data were used for subsequent toxicity analysis.

### 2.3. Sublethal Effects of Tetraniliprole on Life-History Traits of the F_0_ Generation

Uniform-aged third-instar *T. absoluta* larvae were used in life table studies and designated as the F_0_ generation. Sublethal and low lethal concentrations of tetraniliprole (LC_10_ and LC_30_) were applied to assess their effects on the life-history traits of directly exposed individuals. Tomato leaves were treated with the respective concentrations following the procedure outlined in the toxicological bioassay section. After 72 h of exposure, seventy surviving larvae from each treatment group (LC_10_, LC_30_, and control) were individually transferred to separate Petri dishes containing untreated, pesticide-free tomato leaves. Each larva was treated as a single replicate. Moistened cotton wool was used to wrap the petioles of the leaves to prevent wilting and maintain turgor, and leaves were replaced as needed throughout rearing. Larval development and survival were monitored daily. Upon pupation, each individual was transferred to a clean glass tube (1.5 cm diameter × 8 cm height), where pupal duration and survival until adult emergence were recorded. After emergence, one male and one female were paired in a larger glass tube (3 cm diameter × 20 cm height) containing fresh, untreated tomato leaves and a cotton ball soaked in a 10% honey solution as a food source. The leaves with deposited eggs were collected and replaced daily. Adult survival and female fecundity were recorded daily until the death of all individuals. All experimental procedures were conducted under the same controlled environmental conditions.

### 2.4. Transgenerational Effects of Tetraniliprole on Biological Parameters of Subsequent Generations (F_1_ and F_2_)

Transgenerational effects of sublethal and low lethal concentrations of tetraniliprole (LC_10_ and LC_30_, respectively) on F_1_ and F_2_ generations of *T. absoluta* were evaluated using the same experimental protocol used for the F_0_ generation. Eggs laid by F_0_ adults were individually transferred to clean Petri dishes containing untreated tomato leaves and maintained under the same controlled conditions. Each egg served as a single replicate. All subsequent developmental, survival, and reproductive parameters were recorded daily throughout the life cycle. For the F_2_ generation, eggs produced by the F_1_ adults were similarly transferred to untreated tomato leaves and reared using the same procedure. Data collection on developmental duration, survival rates, adult longevity, and fecundity followed the standardized protocol used for the previous generations.

### 2.5. Tetraniliprole-Induced Transgenerational Effects on Developmental and Resistance Genes

The mRNA expression levels of development (juvenile hormone binding protein), reproduction (*Vg*, *VgR*), and resistance genes (*CYP15C1*, *CYP321C40*, *CYP339A1*, *CYP4M116*, *CYP4S55*, *CYP6AB327*, *CYP6AW1*, *CYP9A307v2*) were investigated in response to sublethal and low lethal exposure to tetraniliprole. Total RNA was extracted from third-instar *T. absoluta* larvae at F_0_, F_1_, and F_2_ generations using the RNAsimple Total RNA kit (Tiangen Biotech (Beijing) Co., Ltd., Beijing, China) following the recommended protocol. The RNA quality and quantity were determined by the Bioanalyzer Agilent 2100 (Agilent Technologies, Santa Clara, CA, USA). 1 μg of total RNA was used to synthesize the cDNA using the iScriptTM cDNA Synthesis Kit (Bio-Rad, Berkeley, CA, USA) according to the recommended instructions. RT-qPCR was conducted on a 10 μL total volume of a reaction consisting of 5 μL 2× Kappa SYBR Green I qPCR Mix, 0.2 μL forward and reverse primers (10 μM each), 1 μL of cDNA template, and the remaining volume was nuclease-free water using a CFX Connect TM Real-Time System (Bio-Rad, Berkeley, CA, USA). The thermocycling conditions of each qPCR consist of 95 °C for 45 s, followed by 40 cycles of 95 °C for 15 s, 50–65 °C for 15 s, and 70 °C for 30–60 s. Gene expressions were calculated using the 2^−∆∆Ct^ method [31]. Elongation factor 1 alpha (*EF1α*) and ribosomal protein L28 (*RPL28*) were used as housekeeping genes to normalize the gene expressions. RT-qPCR experiments consist of three biological and three technical replicates. The primers used in the current study are presented in Table 1.

### 2.6. Data and Life Table Analysis

Mortality data were analyzed using probit regression in PoloPlus software version 2.0 (LeOra Software Inc., Berkeley, CA, USA) to estimate the LC_10_, LC_30_, and LC_50_ values of tetraniliprole, along with their corresponding 95% confidence intervals [19]. Life-history data of *T. absoluta* were analyzed based on the age-stage, two-sex life table theory using the TWOSEX-MSChart program (Ver. 07.06.2024) [26,32], which enables comprehensive evaluation of developmental, survival, and reproductive parameters across variable life stages and sexes. Means, variances, and standard errors of the biological and demographic parameters were estimated using a bootstrap resampling procedure with 100,000 iterations [33,34,35]. Statistical differences among treatment groups (LC_10_, LC_30_, and control) and between generations (F_0_, F_1_, and F_2_) within each treatment group were determined using the paired bootstrap test at a significance level of *p* < 0.05. Survival rate, fecundity, life expectancy, and reproductive rate figures were made using SigmaPlot 12.0 (Systat Software Inc., San Jose, CA, USA). 

### 2.7. Population Projection

The TIMING-MSChart program (Ver. 05.07.2024) [36] was used to analyze the population projection according to the standard method [37]. The simulation began with an initial population of 10 *T. absoluta* eggs for each of the control, LC_10_, and LC_30_ cohorts. Under the assumption of no biotic or abiotic suppression, the model projected population growth over 120 days for the F_1_ and F_2_ generations. The net reproductive rate (*R*_0_) confidence interval was generated from 100,000 bootstrap iterations, with its 2.5th and 97.5th percentiles (representing the 2500th and 97,500th sorted values) defining the lower and upper bounds. These percentiles allowed the simulation of population growth to account for all inherent variability and uncertainty [38]. The outcomes of the log-based projections were then visualized using ‘ggplot2’ package of R (Ver. 4.1.3 Vienna, Austria). 

## 3. Results

### 3.1. Toxicity of Tetraniliprole to Tuta absoluta Larvae

The LC_10_, LC_30_, and LC_50_ of tetraniliprole against *T. absoluta* larvae were determined through probit regression analysis. The estimated values were 0.008 mg/L (LC_10_), 0.018 mg/L (LC_30_), and 0.029 mg/L (LC_50_), with corresponding 95% confidence intervals (Table 2). These concentrations, along with a control (acetone), were subsequently used to evaluate their effects on the life table parameters of *T. absoluta* across two consecutive generations (F_1_ and F_2_).

### 3.2. The Sublethal Effects of Tetraniliprole on the Development of the Parental Generation (F_0_)

The sublethal effects of tetraniliprole at LC_10_ and LC_30_ concentrations on the developmental biology of the parental generation (F_0_) of *T. absoluta* are summarized in Table 3. Exposure to both concentrations significantly extended the larval and pupal developmental durations compared to the control group. Specifically, larval development was prolonged by 2.00 and 3.11 days at LC_10_ and LC_30_, respectively, while pupal development increased by 0.91 and 1.53 days. In contrast, adult longevity was significantly reduced in both males and females treated with tetraniliprole, indicating an adverse effect on adult survival. Moreover, a significant reduction in female fecundity was observed, with egg production decreasing by 67.64 and 121.72 eggs/female at LC_10_ and LC_30_, respectively, compared to untreated controls. Additionally, reproductive timing was also disrupted. Both the oviposition period and the adult preoviposition period (APOP) were significantly shortened in treated individuals, further demonstrating the negative impact of tetraniliprole on reproductive capacity and temporal dynamics of *T. absoluta*.

### 3.3. The Sublethal Effects of Tetraniliprole on the Development of the Subsequent Generations (F_1_ and F_2_)

The sublethal effects of tetraniliprole on the development and longevity of *T. absoluta* in the F_1_ and F_2_ generations are detailed in Table 4. At LC_10_, a significant reduction in the duration of egg, larval, and pupal stages was observed relative to the control, suggesting an acceleration of immature development. Conversely, LC_30_ exposure significantly prolonged all three developmental stages across both generations, indicating a dose-dependent developmental delay. When comparing generations, only the egg and larval developmental periods were significantly shorter in LC_30_-exposed individuals in the F_2_ generation compared to F_1_.

Female longevity showed contrasting trends across concentrations and generations. In the F_1_ generation, LC_10_ exposure significantly increased female lifespan by 2.6 days, while LC_30_ exposure reduced it by 3.95 days. However, in the F_2_ generation, no statistically significant difference in female longevity was observed between the control and LC_10_ treatment groups, but LC_30_ reduced female lifespan from 23.80 ± 0.95 to 20.37 ± 0.77 days, confirming the persistent detrimental effects of higher sublethal doses. Male longevity increased significantly under LC_10_ in both generations, but was consistently and significantly reduced at LC_30_ exposure. Interestingly, male longevity was significantly higher in the F_2_ than in the F_1_ within the LC_30_ treatment group. No significant variation in total female longevity was detected between treatment groups or across generations. However, total male longevity was significantly reduced under both LC_10_ and LC_30_ exposures in the F_1_ generation, with no notable differences observed in the F_2_ generation.

### 3.4. The Transgenerational Sublethal Effects of Tetraniliprole on the Progeny Generations (F_1_ and F_2_) of Tuta absoluta

The sublethal effects of tetraniliprole on the life table parameters of *T. absoluta* in the F_1_ and F_2_ generations are presented in Table 5. No significant differences in the net reproductive rate (*R*_0_) were observed between the control and either of the sublethal treatments (LC_10_ or LC_30_), nor were intergenerational variations detected for this parameter, indicating a degree of reproductive stability under sublethal stress. However, both the intrinsic rate of increase (*r*) and the finite rate of increase (*λ*) exhibited concentration-dependent responses. Specifically, LC_10_ exposure significantly elevated both parameters across generations, suggesting a potential hormetic effect. In contrast, exposure to LC_30_ markedly suppressed both r and *λ* values in both generations. The mean generation time (*T*) was also notably affected by tetraniliprole. *T* was significantly shortened under LC_10_ (from 28.50 ± 0.32 to 26.63 ± 0.33 days in the F_1_ generation, and from 29.74 ± 0.33 to 27.00 ± 0.39 days in the F_2_), while LC_30_ increased *T* by 3.96 and 2.65 days in F_1_ and F_2_, respectively.

Fecundity responses were dose- and generation-dependent. LC_10_ significantly enhanced fecundity in both F_1_ and F_2_ generations, while LC_30_ significantly reduced fecundity in the F_1_ generation, with no significant difference in the F_2_. Notably, fecundity under LC_30_ improved by 14.76% in F_2_ compared to F_1_. Regarding the oviposition period, no significant difference was recorded in LC_10_-treated individuals in F_1_. However, in F_2_, oviposition was significantly extended (from 12.17 ± 0.52 to 14.83 ± 0.56 days). An opposite trend was observed for LC_30_-exposed insects, where oviposition was reduced relative to the control. Nonetheless, both LC_10_ and LC_30_ treatments resulted in a notable increase in oviposition period in the F_2_ generation compared to F_1_, highlighting a generational effect. The adult preoviposition period (APOP) was not considerably affected by LC_10,_ but significantly extended under LC_30_ in both generations. A similar pattern was observed for the total preoviposition period (TPOP), which was significantly increased under LC_30_ but reduced under LC_10_.

### 3.5. Age-Stage Specific Survival Rate, Fecundity, and Life Expectancy of Tetraniliprole Exposed Tuta absoluta

**Figure 1:**
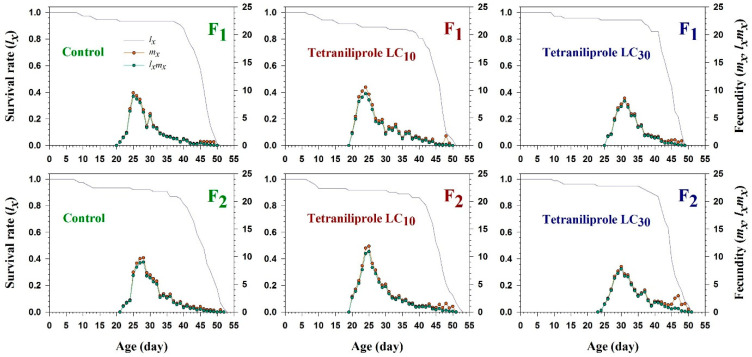
Age-specific survival rate (*l_x_*), age-specific fecundity (*m_x_*), and age-specific maternity (*l_x_m_x_*) of F_1_ and F_2_ generations of *Tuta absoluta* originating from F_0_ individuals exposed to tetraniliprole (LC_10_ and LC_30_).

The age-specific survival rate (*l_x_*), age-specific fecundity (*m_x_*), and the age-specific maternity (*l_x_m_x_*) of *T. absoluta* in the F_1_ and F_2_ generations following tetraniliprole exposure are illustrated in Figure 1. The *m_x_* curves exhibited marked differences in fecundity across generations and treatment levels. At LC_10_, maximum fecundity (0.4–0.6 offspring/female/day) occurred around day 25 in both F_1_ and F_2_ generations. In contrast, at LC_30_, peak fecundity (0.2–0.4) was delayed until approximately day 30, indicating a concentration-dependent effect of tetraniliprole on reproductive timing and output. The trends observed in the maternity function (*l_x_m_x_*) mirrored those of *m_x_*, further highlighting the generational impact of sublethal exposure. However, no significant deviations in the age-specific survival rate (*l_x_*) were observed across treated and control groups in either generation.

**Figure 2:**
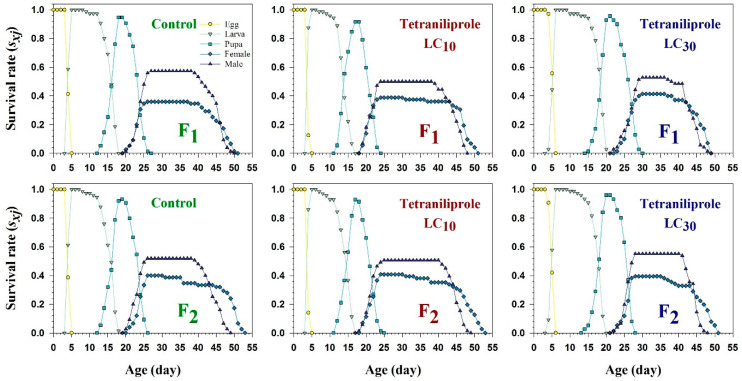
Age-specific survival rate (*s_xj_*) of F_1_ and F_2_ generations of *Tuta absoluta* originating from F_0_ individuals exposed to tetraniliprole (LC_10_ and LC_30_).

**Figure 3:**
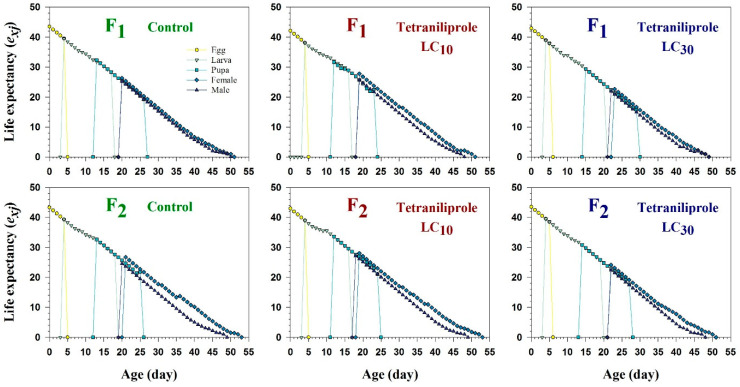
Age-specific life expectancy (*e_xj_*) of F_1_ and F_2_ generations of *Tuta absoluta* originating from F_0_ individuals exposed to tetraniliprole (LC_10_ and LC_30_).

The age-stage-specific survival rate (*s_xj_*), representing the probability that an individual egg survives to age *x* and stage *j*, showed overlapping trends between treatments and control during immature stages (Figure 2). However, *s_xj_* was notably reduced in adult males and females of the F_2_ generation at both LC_10_ and LC_30_. Conversely, larval *s_xj_* values were increased under both concentrations, suggesting a stage-specific response to tetraniliprole exposure. The age-stage-specific life expectancy (*e_xj_*), indicating the expected remaining lifespan of an individual at age *x* and stage *j*, also varied with treatment and generation (Figure 3). Female life expectancy under LC_10_ was 34 days in the F_1_, declining to 27 days at LC_30_. In F_2_, females lived an average of 35 days at LC_10_, while life expectancy decreased to 31 days at LC_30_. These values were slightly lower than the control groups, where life expectancies were 33 and 34 days for F_1_ and F_2_, respectively.

**Figure 4:**
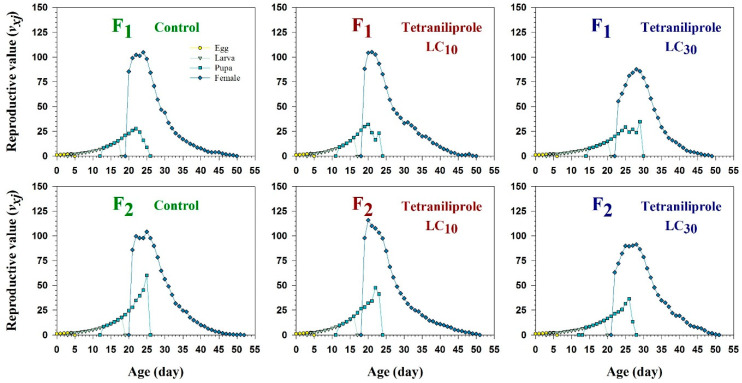
Age-specific reproductive rate (*v_xj_*) of F_1_ and F_2_ generations of *Tuta absoluta* originating from F_0_ individuals exposed to tetraniliprole (LC_10_ and LC_30_).

Age-stage-specific reproductive values (*v_xj_*), representing the future reproductive potential of individuals at each age and stage, were significantly reduced in LC_30_-treated females in both generations compared to controls and LC_10_ treatments (Figure 4). This reduction indicates a transgenerational suppression of reproductive capacity at higher sublethal concentrations.

### 3.6. Population Projection

**Figure 5:**
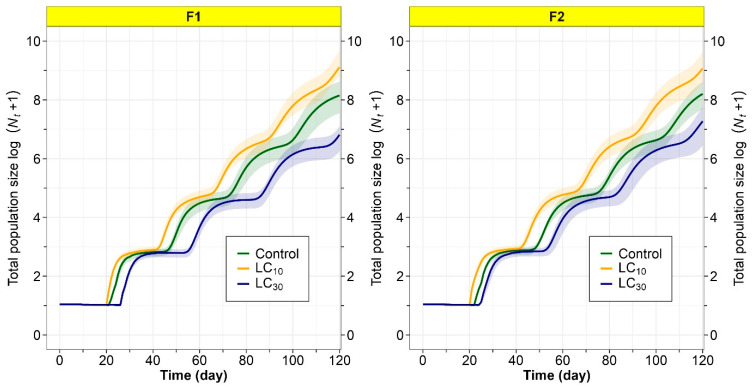
Total population size log (*N_t_ +* 1) of F_1_ and F_2_ generations *Tuta absoluta* originated from F_0_ individuals exposed to the LC_10_ and LC_30_ of tetraniliprole.

The population growth of *T. absoluta* over 120 days (F_1_ and F_2_ generations) for the tetraniliprole treatments (LC_10_, LC_30_) and the control group, including confidence intervals, is shown in Figure 5. Population sizes were greatest in the F_1_ and F_2_ generations after parental exposure to the LC_10_ dose of tetraniliprole, implying a hormetic effect that enhanced population growth. Conversely, population projections for the F_1_ and F_2_ generations following parental exposure to LC_30_ were lower than those of the control, suggesting a suppressive effect at this concentration (Figure 5). This study reveals the concentration-dependent influence of tetraniliprole on *T. absoluta*’s population dynamics across multiple generations.

### 3.7. Transgenerational Effects of Tetraniliprole on Developmental and Resistance Genes

**Figure 6:**
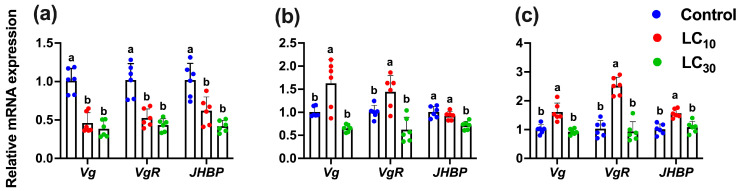
The relative mRNA expression level of development and reproduction-related genes (*Vg*, *VgR*, and *JHBP*) in the parental F_0_ (**a**) and the progeny F_1_ (**b**) and F_2_ (**c**) generations of *Tuta absoluta* after F_0_ treatment with the LC_10_ and LC_30_ of tetraniliprole. The expression level is expressed as the mean (±SE) of the three biological replicates. Letters above the bars represent significant differences at *p* < 0.05 level using one-way analysis of variance with Tukey’s post hoc test (IBM, SPSS Statistics, Armonk, NY, USA, version 29).

**Figure 7:**
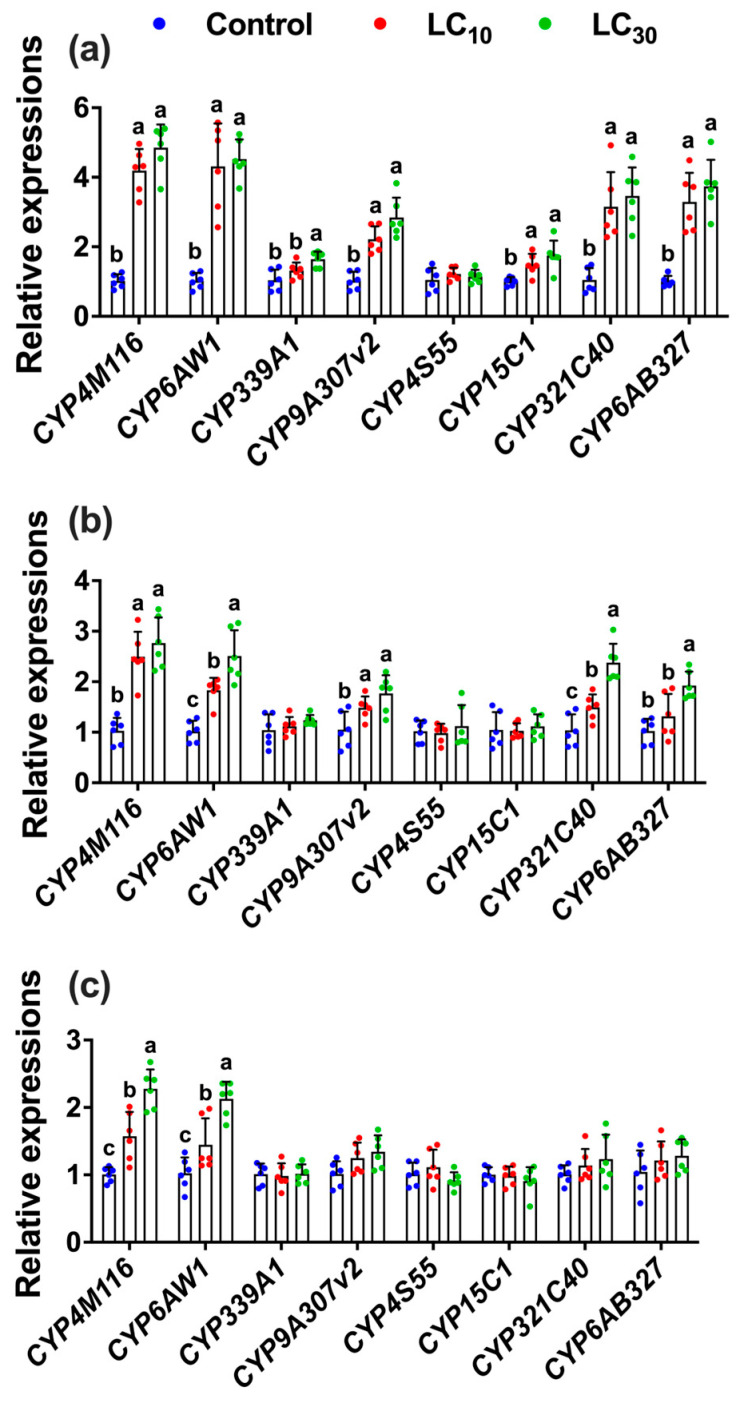
Expressions of P450 genes in the parental F_0_ (**a**) and the progeny F_1_ (**b**) and F_2_ (**c**) generations of *Tuta absoluta* after F_0_ treatment to the LC_10_ and LC_30_ of tetraniliprole. The expression level is expressed as the mean (±SE) of the three biological replicates. Letters above the bars represent significant differences at *p* < 0.05 level using one-way analysis of variance with Tukey’s post hoc test (IBM, SPSS Statistics, Armonk, NY, USA, version 29).

Following the exposure of the parental generation (F0) to sublethal and low-lethal concentrations of tetraniliprole, we analyzed its transgenerational effects on the relative mRNA expression levels of genes linked to development, reproduction, and resistance. These effects were examined in the directly exposed F0 generation as well as in the unexposed progeny generations (F1 and F2) of *T. absoluta* (Figure 6 and Figure 7). Results showed that the expression levels of *Vg*, *VgR*, and *JHBP* were significantly (*Vg*, F_2,17_ = 38.59, *p* < 0.001; *VgR*, F_2,17_ = 27.09, *p* < 0.001; *JHBP*, F_2,17_ = 19.94, *p* < 0.001) reduced in *T. absoluta* directly exposed to the LC_10_ (0.46-, 0.52-, and 0.62-fold) and LC_30_ (0.38-, 0.43-, and 0.41-fold) of tetraniliprole compared to the control (Figure 6a). In the F1 generation, the Vg and VgR were significantly upregulated by 1.62- and 1.44-fold at LC10 (*Vg*, F_2,17_ = 15.55, *p* < 0.001 and *VgR*, F_2,17_ = 14.02, *p* < 0.001), while no effects were noted for LC30 concentration (Figure 6b). In contrast, the expression level of *JHBP* was significantly downregulated by 0.71-fold at LC30 (*JHBP*, F_2,17_ = 13.02, *p* < 0.001), while no effects were observed at LC10 compared to the control. The expression levels of these genes were also upregulated by 1.60-, 2.51-, and 1.56-fold in the LC10-treated individuals (*Vg*, F_2,17_ = 18.92, *p* < 0.001; *VgR*, F_2,17_ = 49.51, *p* < 0.001; *JHBP*, F_2,17_ = 17.06, *p* < 0.001), while no effects were found at the LC_30_-treated group compared to control (Figure 6c).

Further, the expression levels of resistance-related cytochrome P450 genes such as *CYP4M116* (F_2,17_ = 87.38, *p* < 0.001)*, CYP6AW1* (F_2,17_ = 23.13, *p* < 0.001)*, CYP321C40* (F_2,17_ = 17.48, *p* < 0.001)*, CYP6AB327* (F_2,17_ = 29.35, *p* < 0.001)*, CYP9A307v2* (F_2,17_ = 28.83, *p* < 0.001)*, and CYP15C1* (F_2,17_ = 8.57, *p* = 0.003) were all significantly upregulated with fold increases ranging from 1.64- to 4.85-fold in the directly exposed parental generation (F_0_) to the LC_10_ and LC_30_ of tetraniliprole, while *CYP339A1* was upregulated only at LC_30_ as compared to control (Figure 7a). In the F1 generation, the expressions of *CYP4M116* (F_2,17_ = 27.82, *p* < 0.001)*, CYP6AW1* (F_2,17_ = 27.57, *p* < 0.001)*, CYP321C40* (F_2,17_ = 27.62, *p* < 0.001)*, CYP6AB327* (F_2,17_ = 11.40, *p* < 0.001)*,* and *CYP9A307v2* (F_2,17_ = 7.65, *p* = 0.005) were upregulated with fold expressions ranging from 1.48 to 2.76 at the LC_10_ and LC_30_ of tetraniliprole as compared to control (Figure 7b). However, only two P450 genes, such as *CYP4M116* (F_2,17_ = 32.63, *p* < 0.001) and *CYP6AW1* (F_2,17_ = 20.63, *p* < 0.001), were upregulated in the F2 generation with expression ranges from 1.44- to 2.27-fold following parental exposure to the sublethal and low lethal concentrations of tetraniliprole as compared to control (Figure 7c). These results demonstrate that tetraniliprole upregulates resistance-related genes in *T. absoluta*, suggesting enhanced insecticide breakdown and a potential for resistance evolution following sublethal or low-lethal exposure.

## 4. Discussion

This study provides valuable insights into the lethal and sublethal effects of tetraniliprole on *T. absoluta*, a globally important pest of tomato crops. The estimated LC_10_, LC_30_, and LC_50_ values confirm the high toxicity of tetraniliprole to the larval stages of *T. absoluta*, consistent with its known mode of action as a ryanodine receptor modulator in arthropods [39]. These findings support the potential inclusion of tetraniliprole in integrated pest management (IPM) programs targeting *T. absoluta*.

Sublethal (LC10) and low lethal (LC30) exposures significantly impacted developmental and reproductive traits in both the directly exposed parental generation (F_0_) and their offspring (F_1_ and F_2_). In the F_0_ generation, longer larval and pupal stages under sublethal treatments suggest developmental delays caused by physiological stress, likely related to disrupted calcium balance via ryanodine receptor activation [40]. Additionally, earlier research reported that sublethal concentrations of various insecticides, including diamides, caused extended developmental periods in lepidopteran pests [41]. Furthermore, reduced adult lifespan and fecundity in F_0_ indicate a fitness cost among survivors, consistent with previous studies on arthropods (including Lepidopterans) exposed to diamides and other insecticides [17,18,42].

In subsequent generations, responses varied based on both concentration and generation. At LC_10_, a pattern of faster development and higher fecundity indicates a hormetic effect, where low-concentration stress can stimulate physiological processes [43,44]. Conversely, exposure to LC_30_ led to delayed development, decreased fecundity, shorter adult lifespan, and lower reproductive value (*v_xj_*), suggesting transgenerational stress effects or possible epigenetic regulation [45].

Life table parameters clearly reflected these effects. At LC_10_, both the intrinsic rate of increase (*r*) and the finite rate of increase (*λ*) were raised, while at LC_30_, these rates decreased significantly. These patterns are especially important for pest management, as they indicate that sublethal insecticide residues at low concentrations could unintentionally boost population growth, whereas higher sublethal levels may inhibit it [46]. The mean generation time (*T*) was shorter under LC_10_, encouraging faster generational turnover, but longer under LC_30_, which could slow population growth.

Age-stage-specific parameters further supported these findings. Delayed peaks in fecundity, reduced life expectancy (*e_xj_*), and lower reproductive values (*v_xj_*) at LC_30_ highlight disrupted reproductive strategies [32]. Interestingly, increased oviposition periods and population sizes under LC_10_ in F_1_ suggest heightened reproductive effort in response to mild sublethal stress. Sex-specific effects were also prominent. LC_30_ exposure caused a decline in male longevity, potentially impairing mating success and sperm competition [47]. Conversely, increased male longevity at LC_10_ may indicate compensatory mechanisms or decreased mating effort under stress conditions. Overall, these results show that while tetraniliprole is very effective at lethal concentrations, its sublethal effects are complex and can either suppress or promote *T. absoluta* populations depending on the concentration and generation.

*Vg* and *VgR* genes act as key molecular indicators of female reproductive capacity in insects, often reflecting fecundity status under environmental and chemical stressors [48]. While many studies have reported a downregulation of these genes in response to insecticidal pressure, this is frequently seen as a sign of impaired reproduction or endocrine disruption [24,25]. Our study, however, shows a different pattern. Specifically, we observed a significant upregulation of *Vg* and *VgR* expression in insects exposed to the LC_10_ concentration of tetraniliprole, indicating a hormetic response. Hormesis, which is a stimulatory effect at low doses of an otherwise inhibitory agent, may be an adaptive strategy where the organism temporarily boosts reproductive output as a response to mild chemical stress. This reproductive boost was even more noticeable in the F_2_ generation at the LC_30_ level, where *Vg* and *VgR* expression exceeded the levels seen in both F_0_ and F_1_. These findings suggest a transgenerational acclimation effect, possibly driven by epigenetic changes or altered endocrine signaling pathways. In addition to reproductive genes, cytochrome P450 monooxygenases (CYPs) are central to insecticide detoxification and have long been linked to resistance development [30,49]. In our study, *CYP4M116* expression was significantly upregulated in F_0_ after tetraniliprole exposure, reflecting a strong metabolic response in the first generation. However, a decrease in expression levels in F_1_ and F_2_, although still above control levels, may indicate feedback inhibition, reduced selection pressure, or physiological costs across generations. These patterns highlight the dynamic nature of gene-environment interactions that shape resistance phenotypes over time.

In contrast, *CYP6AB327* and *CYP6AW1* showed sustained upregulation across LC_10_ and LC_30_ treatments and generations, indicating a more consistent and potentially heritable metabolic resistance mechanism. The involvement of these genes aligns with their known roles in detoxifying various insecticides, including spinosad, diamides, and neonicotinoids [50,51]. However, the absence of significant expression in other P450 genes such as *CYP4S55*, *CYP9A307v2*, and *CYP15C1* highlights that detoxification pathways are gene-specific and selective, likely influenced by the chemical structure of the insecticide, dosage, and exposure duration.

These findings agree with an increasing amount of evidence suggesting that resistance can develop from the activation of common detoxification genes due to the widespread use of chemical insecticides [1,50]. This overlap in metabolic pathways presents a significant risk to sustainable pest management, as it can weaken rotation strategies if cross-resistance genes are not accurately identified and monitored. Overall, our results lay the groundwork for understanding the complex transgenerational effects of tetraniliprole on *T. absoluta* and their impact on population management. Future research should investigate these dynamics under continuous exposure across multiple generations to better reflect field conditions. Such studies would help determine whether the observed hormetic response persists, diminishes, or increases over time, and how including generational mortality in population growth models might affect the long-term dynamics of pest populations under ongoing insecticide use.

## 5. Conclusions

In conclusion, tetraniliprole shows dual concentration-dependent transgenerational effects on *T. absoluta*, causing hormesis at LC_10_ but suppression at LC_30_. These sublethal effects, driven by changes in gene expression, emphasize a significant risk of population resurgence and should be considered in resistance management strategies.

## Figures and Tables

**Table 1:** The primers used for RT-qPCR and dsRNA synthesis.

Primer Name	Forward Sequence	Reverse Sequence
*JHBP*	CCCATTAACCATGCCACAGG	TGAAGCTTTTCCTGGTGTGTC
*Vg*	TGGTACGTGGTTATGCAGGA	TACTTCGACACTGGGGGTTC
*VgR*	ATCTTTGTCCGGACCACACT	CGTCTGCACAATCTGTCTCG
*CYP4M116*	GACGCCAACTTTCCACTTCAAC	GCCCATCGCTGTTTCGCATA
*CYP6AW1*	GCCTTGAAACATCAGCCACAAC	GTCAATCCGTCGTGCTTACTCA
*CYP339A1*	TCTCGCTTCACCTCGTCCTG	CGAACGGCAGAACCATAGACTC
*CYP9A307v2*	AAAGGTTCGTGGGCAGATTCG	TCGTTCAGGAAGTCTCGGTGAT
*CYP4S55*	GGTTCCACGAGAGCATCTATTCA	CGAGAGCACCACCTCAACATC
*CYP15C1*	GCAGCAGGAGATAGATGAAGTCA	CACGGAGGATATGCGAAGAGTT
*CYP321C40*	GGAATGAGATACGCACGACTACA	CACCGCTTGCTTGCTGTACT
*CYP6AB327*	AAGGTGCTCTAGTGGGAGAATCT	AATCCTGCGGCGAAGAATACAA
*EF1α*	GAAGCCTGGTATGGTTGTCGT	GGGTGGGTTGTTCTTTGTG
*RPL28*	TCAGACGTGCTGAACACACA	GCCAGTCTTGGACAACCATT

**Table 2:** Toxicity of tetraniliprole against third-instar *Tuta absoluta* after 48 h exposure.

Treatments	Slope ± SE ^a^	LC_10_ mg/L (95% CL) ^b^	LC_30_ mg/L (95% CL) ^b^	LC_50_ mg/L (95% CL) ^b^	χ^2^ (*df*) ^c^	*p*-Value
Tetraniliprole	2.369 ± 0.216	0.008 (0.006–0.011)	0.018 (0.014–0.021)	0.029 (0.025–0.034)	7.035 (19)	0.994

^a^ Standard error; ^b^ 95% confidence intervals; ^c^ Chi-square value (*χ*^2^) and degrees of freedom (*df*) calculated by PoloPlus 2.0.

**Table 3:** Duration of various developmental stages and some reproductive parameters of parental generation (F_0_) of *Tuta absoluta* treated with LC_10_ and LC_30_ of tetraniliprole.

Parameters	Control		Tetraniliprole LC_10_		Tetraniliprole LC_30_	
	*n*	Mean ± SE	*n*	Mean ± SE	*n*	Mean ± SE
Larva (days)	74	11.72 ± 0.14 ^c^	69	13.72 ± 0.12 ^b^	69	14.83 ± 0.18 ^a^
Pupa (days)	72	7.21 ± 0.08 ^c^	68	8.12 ± 0.08 ^b^	69	8.74 ± 0.07 ^a^
Female longevity (days)	31	23.06 ± 0.54 ^a^	30	16.47 ± 0.88 ^b^	27	13.89 ± 0.63 ^c^
Male longevity (days)	41	21.12 ± 0.35 ^a^	38	16.21 ± 0.36 ^b^	42	13.64 ± 0.23 ^c^
Fecundity (eggs/female)	31	192.87 ± 6.54 ^a^	30	125.23 ± 5.11 ^b^	27	71.15 ± 3.52 ^c^
Oviposition days (days)	31	13.00 ± 0.38 ^a^	30	8.73 ± 0.50 ^b^	27	5.96 ± 0.44 ^c^
Adult preoviposition period (days)	31	1.19 ± 0.07 ^b^	30	2.17 ± 0.08 ^a^	27	2.37 ± 0.09 ^a^

Standard errors were estimated by using the bootstrap technique with 100,000 resamples. Differences were compared using the paired bootstrap test (*p* < 0.05). Means within a row followed by different lowercase letters indicate significant differences among the treatments.

**Table 4:** Duration of various developmental stages of two subsequent *Tuta absoluta* generations (F_1_ and F_2_), whose parents (F_0_) were treated with LC_10_ and LC_30_ of tetraniliprole.

Durations (Days)	Generations	Control		Tetraniliprole LC_10_		Tetraniliprole LC_30_	
		*n*	Mean ± SE	*n*	Mean ± SE	*n*	Mean ± SE
Egg	F_1_	75	4.41 ± 0.06 ^bA^	72	4.13 ± 0.04 ^cA^	70	5.53 ± 0.07 ^aA^
	F_2_	75	4.39 ± 0.06 ^bA^	71	4.14 ± 0.04 ^cA^	76	5.33 ± 0.07 ^aB^
Larva	F_1_	71	11.80 ± 0.16 ^bA^	68	10.24 ± 0.16 ^cA^	67	13.12 ± 0.14 ^aA^
	F_2_	70	11.76 ± 0.17 ^bA^	66	10.65 ± 0.19 ^cA^	73	12.71 ± 0.14 ^aB^
Pupa	F_1_	70	7.43 ± 0.10 ^bA^	64	6.84 ± 0.10 ^cA^	66	7.88 ± 0.15 ^aA^
	F_2_	69	7.42 ± 0.10 ^bA^	65	6.63 ± 0.09 ^cA^	72	7.82 ± 0.06 ^aA^
Total Preadult	F_1_	70	23.64 ± 0.19 ^bA^	64	21.23 ± 0.18 ^cA^	66	26.52 ± 0.23 ^aA^
	F_2_	69	23.49 ± 0.19 ^bA^	65	21.48 ± 0.20 ^cA^	72	25.86 ± 0.17 ^aB^
Female longevity	F_1_	27	23.19 ± 0.53 ^bA^	28	25.79 ± 0.68 ^aA^	29	19.24 ± 0.61 ^cA^
	F_2_	30	23.80 ± 0.95 ^aA^	29	25.52 ± 0.89 ^aA^	30	20.37 ± 0.77 ^bA^
Male longevity	F_1_	43	21.30 ± 0.31 ^bA^	36	23.28 ± 0.36 ^aA^	37	17.35 ± 0.26 ^cB^
	F_2_	39	21.46 ± 0.30 ^bA^	36	23.72 ± 0.20 ^aA^	42	18.52 ± 0.22 ^cA^
Total female longevity	F_1_	27	46.33 ± 0.65 ^aA^	28	46.79 ± 0.77 ^aA^	29	45.69 ± 0.60 ^aA^
	F_2_	30	47.77 ± 1.06 ^aA^	29	47.07 ± 1.03 ^aA^	30	46.17 ± 0.78 ^aA^
Total male longevity	F_1_	43	45.26 ± 0.41 ^aA^	36	44.69 ± 0.42 ^abA^	37	43.92 ± 0.43 ^bA^
	F_2_	39	44.59 ± 0.42 ^aA^	36	45.14 ± 0.33 ^aA^	42	44.43 ± 0.26 ^aA^

Standard errors were estimated by using the bootstrap technique with 100,000 resampling. Difference was compared using the paired bootstrap test (*p* < 0.05). Significantly different means within a row are denoted by different lowercase letters, while the means within a column followed by different uppercase letters indicate significant differences between two subsequent generations, F_1_ and F_2_.

**Table 5:** Reproduction and life table parameters of two subsequent (F_1_ and F_2_) generations of *Tuta absoluta*, whose parents (F_0_) were treated with LC_10_ and LC_30_ of tetraniliprole.

Parameters	Generations	Control	Tetraniliprole LC_10_	Tetraniliprole LC_30_
		Mean ± SE	Mean ± SE	Mean ± SE
Net reproductive rate (*R*_0_) (offspring/individual)	F_1_	70.29 ± 10.94 ^aA^	86.24 ± 13.20 ^aA^	64.41 ± 9.34 ^aA^
	F_2_	80.68 ± 11.93 ^aA^	94.38 ± 13.67 ^aA^	72.00 ± 10.53 ^aA^
Intrinsic rate of increase (*r*) (day^−1^)	F_1_	0.1492 ± 0.0061 ^bA^	0.1674 ± 0.0064 ^aA^	0.1272 ± 0.0047 ^cA^
	F_2_	0.1476 ± 0.0055 ^bA^	0.1684 ± 0.0061 ^aA^	0.1320 ± 0.0049 ^cA^
Finite rate of increase (*λ*) (day^−1^)	F_1_	1.1609 ± 0.0070 ^bA^	1.1822 ± 0.0076 ^aA^	1.1356 ± 0.0054 ^cA^
	F_2_	1.1591 ± 0.0063 ^bA^	1.1835 ± 0.0072 ^aA^	1.1412 ± 0.0056 ^cA^
Mean generation time (*T*) (days)	F_1_	28.50 ± 0.32 ^bB^	26.63 ± 0.33 ^cA^	32.76 ± 0.31 ^aA^
	F_2_	29.74 ± 0.33 ^bA^	27.00 ± 0.39 ^cA^	32.39 ± 0.35 ^aA^
Fecundity (*F*) (eggs/female)	F_1_	195.26 ± 3.99 ^bA^	221.75 ± 8.83 ^aA^	155.48 ± 4.41 ^cB^
	F_2_	201.70 ± 8.56 ^bA^	231.07 ± 6.12 ^aA^	182.40 ± 6.53 ^bA^
Ovipositon days (*O_d_*) (days)	F_1_	12.96 ± 0.33 ^aA^	12.93 ± 0.53 ^aB^	10.10 ± 0.45 ^bB^
	F_2_	12.17 ± 0.52 ^bA^	14.83 ± 0.56 ^aA^	13.00 ± 0.61 ^bA^
Adult preoviposition period (APOP) (days)	F_1_	1.26 ± 0.09 ^bA^	1.18 ± 0.07 ^bA^	2.24 ± 0.08 ^aA^
	F_2_	1.27 ± 0.08 ^bA^	1.21 ± 0.08 ^bA^	2.00 ± 0.09 ^aA^
Total preoviposition period (TPOP) (days)	F_1_	24.41 ± 0.27 ^bB^	22.18 ± 0.28 ^cA^	28.69 ± 0.31 ^aA^
	F_2_	25.23 ± 0.27 ^bA^	22.76 ± 0.33 ^cA^	27.80 ± 0.29 ^aB^

Standard errors were estimated by using the bootstrap technique with 100,000 resampling. Difference was compared using the paired bootstrap test (*p* < 0.05). Significantly different means within a row are denoted by different lowercase letters, while the means within a column followed by different uppercase letters indicate significant differences between two subsequent generations, F1 and F2.

## Data Availability

The original contributions presented in this study are included in the article. Further inquiries can be directed to the corresponding authors.
